# OUTCOMES ASSOCIATED WITH PARTICIPATION IN THE ILLINOIS WORKPLACE WELLNESS STUDY

**DOI:** 10.1093/geroni/igae098.3240

**Published:** 2024-12-31

**Authors:** Laura Payne, Do Yun Kim, Jaesung An, Megan Janke, Elizabeth Orsega-Smith, Julie Bobitt, Neha Gothe

**Affiliations:** University of Illinois Urbana-Champaign, Urbana, Illinois, United States; University of Illinois Urbana-Champaign, Champaign, Illinois, United States; California State University East Bay, Hayward, California, United States; Berry College, Rome, Georgia, United States; University of Delaware, Newark, Delaware, United States; University of Illinois Chicago, Chicago, Illinois, United States; Northeastern University, Boston, Massachusetts, United States

## Abstract

Workplace wellness (WW) programs assert health benefits; however, most available evidence is based on observational studies comparing participants to non-participants (Reif et al., 2020). Experiences and outcomes associated with WW program participation may vary by the age. This study examined factors that shaped participation among younger (under 50) and older employees (age 50+) in a workplace wellness program. Using data from the Illinois WW Study (N=4,834), we examined several health behaviors (physical activity, smoking, alcohol use), participation rates, and presenteeism. Participants were randomly assigned to treatment and control groups; they completed a baseline survey and annual follow-up surveys for three years. Physical activity (PA) was measured with three questions (compared to other people your age are you more active, about the same, less active? Are you trying to increase your PA? In the last 12 months, has your doctor told you to increase your PA?). Smoking and alcohol use were assessed with the number of days smoked/drank in a week and the number of smokes/drinks/day. Presenteeism was measured with the 6-item Stanford presenteeism scale. The 50 and over age group scored significantly higher (M = 3.31, SD = 0.63) than the under 50 group (M = 3.07, SD = 0.70) on the composite health behavior score. For all four study periods (i.e., fall 2016, spring 2017, fall 2017, and spring 2018), there was no significant association between age and workplace wellness program participation. Results show no significant difference in absenteeism between the control and treatment group over the study period.

